# Molecular genetic characterization of *Cryptosporidium* and *Cystoisospora* protozoan infections in cats from large cities of Kazakhstan

**DOI:** 10.3389/fpara.2025.1608542

**Published:** 2025-07-14

**Authors:** Lyudmila Lider, Rabiga Uakhit, Nurassyl Manapov, Valentina Yerzhanova, Alexandr Andreyev, Ainura Smagulova, Carlos Hermosilla, Vladimir Kiyan

**Affiliations:** ^1^ Laboratory of Parasitology, Department of Veterinary Medicine, S. Seifullin Kazakh Agrotechnical Research University, Astana, Kazakhstan; ^2^ Laboratory of Biodiversity and Genetic Resources, National Center for Biotechnology, Astana, Kazakhstan; ^3^ Institute of Parasitology, Justus Liebig University Giessen, Giessen, Germany

**Keywords:** *Cryptosporidium parvum*, gp60 subtyping, molecular epidemiology, zoonotic parasites, Kazakhstan

## Abstract

**Introduction:**

*Cryptosporidium* spp. and *Cystoisospora* spp. are significant unicellular parasites that cause gastrointestinal infections in both humans and animals globally. Among these, *Cryptosporidium felis* and *Cystoisospora felis* are particularly important for feline health and pose potential zoonotic risks, especially for individuals with compromised immune systems. Kazakhstan, characterized by its diverse climate zones and an increasing population of pets, provides an excellent context for studying the epidemiology and genetic diversity of these parasites. In Kazakhstan, the mandatory registration of pets offers a valuable opportunity to explore the distribution and molecular characteristics of these parasites. This study focuses on the prevalence, genetic diversity, and zoonotic potential of *Cryptosporidium* and *Cystoisospora* from companion and shelter cats across five major cities in Kazakhstan.

**Methods:**

Overall, from five cities, 1301 fecal samples were collected and studied. Samples were study by direct modified Sheather’s flotation technique was applied using a sugar solution. Samples were screened using the 18S rRNA gene for Cryptosporidium and the ITS-1 gene for Cystoisospora. Nucleotide sequences were aligned with the MUSCLE multiple sequence alignment program. Phylograms were constructed with the MEGA11 software using the Maximum Likelihood (ML) method.

**Results and discussion:**

In total, we examined 1,301 fecal samples and found that 31 (2.4%) contained *Cryptosporidium* spp., including 10 identified as *Cryptosporidium felis*. Additionally, 121 samples (9.3%) tested positive for *Cystoisospora felis*. The studied *Cryptosporidium parvum* isolates obtained in this study belong to subtype IIdA15G1, which is dominant and clusters well with previously reported sequences from different countries on the gp60 gene. Shelter cats are more susceptible to these parasites, with a prevalence of 3.1% for *Cryptosporidium* and a notably higher rate of 19.0% for *Cystoisospora*. In contrast, companion cats showed lower rates, at 1.6% for *Cryptosporidium* and 5.1% for *Cystoisospora*. Our findings identified the species *Cystoisospora felis*, *Cryptosporidium parvum*, and *Cryptosporidium felis*, with a determined subtype of XIXa.

## 1 Introduction

*Cryptosporidium* spp. and *Cystoisospora* spp. are unicellular parasites of significant medical and veterinary importance, causing gastrointestinal infections in a wide range of hosts, including humans, domestic animals, and wildlife (Lappin, 2005; Morelli et al., 2021; Guo et al., 2022). These pathogens are particularly concerning due to their ability to induce severe diarrheal disease, their environmental resilience, and, in some cases, their zoonotic potential (Santin, 2020; Baptista et al., 2021). Among the affected species, domestic cats (*Felis catus*) serve as important hosts for *Cryptosporidium felis (C. felis)* and *Cystoisospora felis*, which can lead to clinical illness in feline populations and pose potential risks to human health, especially in immunocompromised individuals (Santin, 2020).

Enteric unicellular parasites are found worldwide and are primarily maintained in nature through fecal-oral transmission, which means that more cases tend to arise in crowded and unsanitary environments (Certad et al., 2017; Relat and O’Connor, 2020). *Cryptosporidium* spp. oocysts are immediately infectious upon being excreted by the host, whereas *Cystoisospora* spp. must first sporulate outside the host before they can spread (Matsubayashi et al., 2011; Dubey, 2018). *Cryptosporidium* is transmitted through the fecal-oral route via both direct and indirect means. Direct transmission occurs when an individual ingests oocysts from the feces of an infected host, while indirect transmission happens through the consumption of water or food contaminated with these oocysts. Over 20 different *Cryptosporidium* species have been linked to human cryptosporidiosis, with the most prevalent being *C. hominis*, *C. parvum*, *C. meleagridis*, *C. felis*, and *C. canis* (Checkley et al., 2015). Among these, *C. felis* primarily infects cats, making it a species adapted specifically to its host (Feng et al., 2018; Zahedi and Ryan, 2020). However, infections in humans from *C. felis* are frequently observed in developing nations (Caccio et al., 2002; Xiao, 2010; Cieloszyk et al., 2012; Beser et al., 2015; de Lucio et al., 2016), and there have been documented cases of zoonotic transmission of *C. felis* between pet cats and their owners (Power et al., 2011).

Similarly, *Cystoisospora* follows a comparable transmission pattern, but its oocysts must sporulate in the environment to become infectious (Matsubayashi et al., 2011; Dubey, 2018). Once ingested, its oocysts release sporozoites that invade intestinal cells, resulting in mucosal inflammation and malabsorptive diarrhea. *Cystoisospora felis* and *Cystoisospora rivolta* are obligate intracellular coccidian parasites that primarily infect domestic and wild felids (Shafiei et al., 2016). Current evidence suggests that *Cystoisospora felis* and *C. rivolta* are not transmissible to humans, with human cystoisosporiasis exclusively caused by the morphologically similar but genetically distinct *C. belli* (Lindsay, 2019; Guzmán-Lara et al., 2020). Although zoonotic risks appear negligible, the morphological similarity between *Cystoisospora* species warrants continued differentiation from truly zoonotic coccidian-like *Cryptosporidium* (Li et al., 2020).

In Kazakhstan, the epidemiology of these parasites in feline populations remains understudied, despite the country’s diverse climatic conditions and the increasing number of companion animals. Since September 2023, Kazakhstan has implemented a mandatory companion animal registration system, which reported approximately 88,514 registered cats by March 2025 (TAÑBA in digits, 2025). The aim of this study is to genetically characterize *Cryptosporidium* spp. and *Cystoisospora felis* infections in domestic and shelter cats across major cities in Kazakhstan, using 18S rRNA, gp60, and ITS-1 (Power et al., 2011; Shafiei et al., 2016; Rojas-Lopez et al., 2020; Koseoglu et al., 2022) gene markers. This study aims to determine the prevalence and distribution of these protozoan infections in feline populations across five major cities (Astana, Almaty, Shymkent, Oral, and Kostanay).

## 2 Materials and methods

### 2.1 Sample collection and analysis

**Figure 1:**
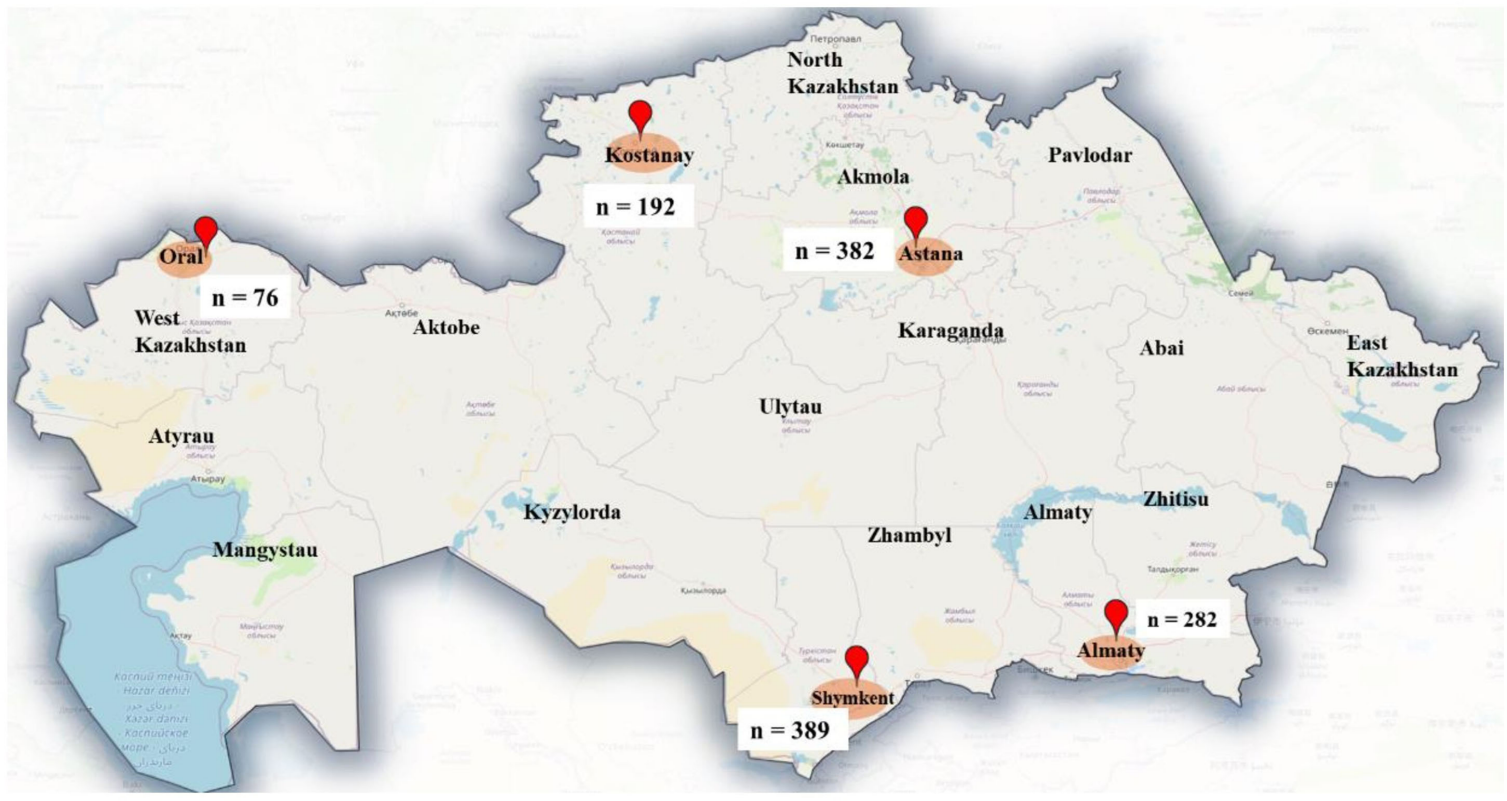
Map showing the geographical locations of cities where cat feces samples were collected.

The distribution of sampling sites suggests a broad geographic coverage, spanning the western, northern, central, southern, and southeastern regions of Kazakhstan. This ensures a representative dataset for studying the prevalence of parasitic infections in cats across diverse climatic and ecological zones (**Figure 1**).

Overall, from five cities, 1301 samples were collected and studied. The samples were collected either during visits to one of the local veterinary clinics or upon the rescue of strays at shelters involved in the study. The fecal samples were sent to the Parasitology Laboratory of S. Seifullin Kazakh Agro Technical Research University in Astana for analysis within a span of 1 to 3 days, using coolers for transport. Sample collection occurred during the following intervals: from September 2023 to January 2025.

All fecal samples were visually inspected for the presence of cestode proglottids, after which the direct modified Sheather’s flotation technique was applied using a sugar solution (specific gravity: 1.3) as the flotation medium for worm eggs and coccidian oocysts (Kassai et al., 2013). The identification of the parasitic fecal stages relied on their morphological features (Kassai et al., 2013).

### 2.2 DNA extraction

Genomic DNA was isolated from approximately 150-200 mg of fecal matter using the QIAamp DNA Stool Mini Kit (QIAGEN, Hilden, Germany) following the instructions provided by the manufacturer; samples mixed with InhibitEX buffer were incubated for 10 minutes at 95°C. The extracted and purified DNA samples (50 µl) were stored at −20°C until further molecular analyses were performed.

### 2.3 Molecular identification

The molecular identification of *Cryptosporidium* and *Cystoisospora* was conducted by extracting DNA and amplifying with specific target genes from fecal samples using a thermocycler (Eppendorf Mastercycler, Hamburg Germany) (**Table 1**). Samples were screened using the 18S rRNA gene for *Cryptosporidium* and the ITS-1 gene for *Cystoisospora* (Power et al., 2011; Shafiei et al., 2016). The nested PCR was performed for the gp60 gene for *C. felis* (Rojas-Lopez et al., 2020; Koseoglu et al., 2022; Yun et al., 2023) to identify their subtypes. A negative template control sample (nuclease-free water) was included in each PCR to verify the absence of contamination with the PCR reaction mixture. Agarose gels (1.5%) were prepared in 1× TAE solution with 8 ng/µL ethidium bromide (Sigma, E1510). Electrophoresis was performed using 10 μL PCR products with a DirectLoad 100 bp Low ladder ready-to-use (Sigma, D3687-1VL) for 50 min at 120 V. The PCR-amplified target gene fragment was purified using a QIAquick PCR Purification Kit, (QIAGEN, Germany, Cat.: 28106), following the manufacturer’s protocols. Sequencing was performed according to the manual for Seq Studio Genetic Analyzer (Thermo Fisher Scientific Applied Biosystems). The resulting nucleotide sequences were visually checked by the Bioedit program version 7.0. The nucleotide sequences of the studied species were compared with other sequences in the NCBI gene bank database by using the BLAST options (http://blast.ncbi.nlm.nih.gov). The nucleotide sequences of the studied species were deposited in NCBI GenBank database.

**Table 1:** Primer sequences and PCR conditions used for the molecular identification.

Target species	Gene	Primer nucleotide sequences (5’-3’)	Size (bp)	Cycling conditions	Ref.
*Cryptosporidium*	18S rRNA	F: AGTGACAAGAAATAACAATACAGG	295	2m/96°C, 40 cycles (30s/94°C, 30s/60°C, 60s/72°C), 7m/72°C	(Shafiei et al., 2016)
		R: CCTGCTTTAAGCACTCTAATTTTC			
*Cryptosporidium* *felis*	gp60	F1: TTTCCGTTATTGTTGCAGTTGCA	1,200	PCR1 and PCR2: 4m/95°C, 35 cycles (30s/95°C, 30s/55°C, 90s/72°C), 7m/72°C	(Koseoglu et al., 2022)
		R1: ATCGGAATCCCACCATCGAAC			
		F2: GGGCGTTCTGAAGGATGTAA	900		
		R2: CGGTGGTCTCCTCAGTCTTC			
*Cystoisospora*	ITS-1	F1: CCGTTGCTCCTACCGATTGAGTG	450	PCR1 and PCR2: 60s/94°C, 40 cycles (10s/98°C, 15s/62°C, 60s/68°C), 5m/68°C	(Power et al., 2011)
		R1: GCATTTCGCTGCGTCCTTCATCG			
		F2: GATCATTCACACGTGGCCCTTG			
		R2: GACGACGTCCAAATCCACAGAGC			

### 2.4 Phylogenetic analysis

Nucleotide sequences were aligned with the MUSCLE multiple sequence alignment program for 18s rRNA, gp60 for *Cryptosporidium* species and ITS-1 genes for *Cystoisospora* isolates. Phylograms were constructed with the MEGA11 software (Tamura et al., 2021) using the Maximum Likelihood (ML) method.

### 2.5 Statistical analysis

The frequency of gastrointestinal unicellular parasite isolates detected from fecal samples was statistically compared using the chi-square test, with a 95% confidence interval. The P-value was calculated, with statistical significance established at P < 0.05.

## 3 Results

Among the 1301 cats examined, 912 (70.1%) were classified as companion cats, whereas 389 (29.9%) originated from shelters. The distribution of cats sampled across various cities included Shymkent (389), Astana (382), Almaty (282), Kostanay (192), and Oral (76) (**Table 2**).

**Table 2:** Coprological analyses of the fecal samples of cats.

Parameter	No. of tested	No. of isolates					
		*Cryptosporidium*	CI (95%)	*p*-value	*Cystoisospora*	CI (95%)	*p*-value
City				<0.01			<0.01
Astana	382	11	2.9 (1.5–5.1)		31	8.1 (5.6–11.3)	
Almaty	282	8	2.9 (1.2–5.6)		8	2.9 (1.2–5.6)	
Shymkent	369	4	1.1 (0.3–2.8)		62	16.8 (13.1–21.0)	
Oral	76	5	6.6 (2.2–14.7)		14	18.4 (10.4–29.0)	
Kostanay	192	3	1.6 (0.3–4.6)		6	3.1 (1.1–6.7)	
Season				<0.05			<0.06
Spring	173	5	2.9 (1.0 – 6.6)		31	17.9 (12.5–24.4)	
Summer	272	5	1.8 (0.6 – 4.2)		32	11.8 (8.2 – 16.2)	
Autumn	624	12	1.9 (1.0 – 3.3)		34	5.4 (3.8 – 7.5)	
Winter	232	9	3.9 (1.8 – 7.2)		24	10.3 (6.7 – 15.0)	
Sex				<0.04			<0.04
Male	559	14	2.5 (1.4 – 4.2)		54	9.7 (7.3 – 12.4)	
Female	655	17	2.6 (1.5 – 4.1)		67	10.2 (8.0 – 12.8)	
N/A	87	–	–		–	–	
Living condition				<0.2			<0.2
Companion	912	15	1.6 (0.9 – 2.7)		47	5.1 (3.8 – 6.8)	
Shelter	389	12	3.1 (1.6 – 5.3)		74	19.0 (15.2–23.3)	
Age class				<0.005			<0.005
1-7 month	322	16	4.5 (2.9 – 8.0)		35	10.9 (7.7 – 14.8)	
>7 month to 3 years	399	10	2.5 (1.2 – 4.6)		31	7.8 (5.3 – 10.9)	
>3 years	387	5	1.3 (0.4 – 2.3)		29	7.5 (5.1 – 10.6)	
N/A	193	–	–		–	–	
Total	1301	31	2.4 (1.6 – 3.4)		121	9.3 (7.8 – 11.0)	

When examining the gender ratio, the results revealed 655 males (54.0%) and 559 females (46.0%). Approximately a quarter of the cats (N = 387; 38.9%) were older than three years, 322 (32.1%) were aged between 1 and 7 months, and 399 (40.1%) fell within the range of 7 months to three years. Overall, the findings indicated that gastrointestinal protozoan parasites were detected in 152 out of 1301 (14.44%; 95% CI 1.0–13.55) fecal samples from the cats examined. Multiple protozoa parasites were detected in nine fecal samples, they were co-infected with *Cystoisospora* and *Cryptosporidium* (data not shown).

**Figure 2:**
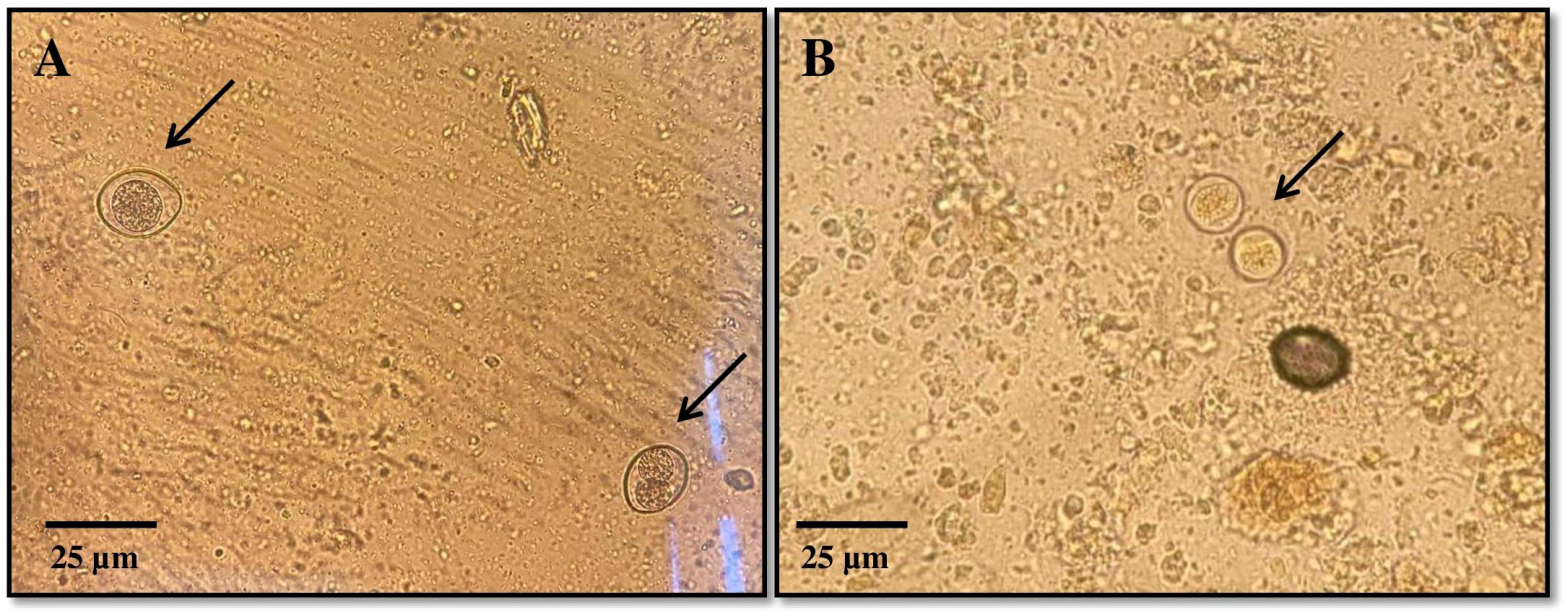
Representative images of *Cystoisospora* spp. oocysts detected during coprological analysis: **(A)** – *Cystoisospora felis*, **(B)** – *Cystoisospora rivolta*.

The coproscopic analysis revealed the presence of *Cystoisospora felis* in 94 out of 1301 samples and *C. rivolta* in 26 out of 1301 samples. Additionally, *Cryptosporidium* spp. was detected in 31 out of 1301 samples. As shown in **Figure 2**, observed *C. felis* large ovoid oocysts measuring 32-53 × 26-43 µm, light yellow or light brown. *C. rivolta* oval or ovoid, size 23-29 × 20-26 µm, light brown, may be colorless.

The isolates of *C. felis* identified through the 18S rRNA gene were sequenced using the gp60 gene to determine the subtype of *C. felis*. Sequence analysis of the gp60 gene revealed that 2 out of the 31 isolates were successfully identified as *C. felis* and revealed that isolates in the present study relate to subtype XIXa. Meanwhile, 12 out of 31 isolates were confirmed as *C. parvum* using the 18S rRNA gene. The 121 samples tested positive for *Cystoisospora* sp., with 10 of them identified as *C. felis* (**Table 3**).

**Table 3:** Data availability of identified species, subtype and GenBank accession number.

Species	Sample ID	Gene	Accession number	Subtype	Geolocation
*Cryptosporidium parvum*	CRpJ13-1	18S rRNA	PV195225		Shymkent
*Cryptosporidium parvum*	CRpJ13-3	18S rRNA	PV195226		Shymkent
*Cryptosporidium parvum*	CRpJ13-7	18S rRNA	PV195227		Shymkent
*Cryptosporidium parvum*	CRpJ13-12	18S rRNA	PV195228		Shymkent
*Cryptosporidium parvum*	CRpJ13-13	18S rRNA	PV195229		Shymkent
*Cryptosporidium parvum*	CRpJ13-15	18S rRNA	PV195230		Shymkent
*Cryptosporidium parvum*	CRpJ13-21	18S rRNA	PV196854		Shymkent
*Cryptosporidium parvum*	CRpJ13-23	18S rRNA	PV196855		Shymkent
*Cryptosporidium parvum*	CRpA4	18S rRNA	PV262383		Astana
*Cryptosporidium parvum*	CRpF32	18S rRNA	PV262384		Astana
*Cryptosporidium parvum*	CRpF37	18S rRNA	PV262385		Astana
*Cryptosporidium parvum*	CRpS3	18S rRNA	PV262386		Astana
*Cryptosporidium felis*	CRfU-6	18S rRNA	PV262388		Oral
*Cryptosporidium felis*	CRfF-35	18S rRNA	PV262389		Astana
*Cryptosporidium felis*	CrypfU-6	gp60	PV221954	XIXa	Oral
*Cryptosporidium felis*	CrypfF-35	gp60	PV221955	XIXa	Astana
*Cystoisospora felis*	CystoAs1	ITS-1	PV259753		Astana
*Cystoisospora felis*	CystoAs2	ITS-1	PV259754		Astana
*Cystoisospora felis*	CystoAl1	ITS-1	PV259755		Almaty
*Cystoisospora felis*	CystoAl2	ITS-1	PV259756		Almaty
*Cystoisospora felis*	CystoSh1	ITS-1	PV259766		Shymkent
*Cystoisospora felis*	CystoSh2	ITS-1	PV259767		Shymkent
*Cystoisospora felis*	CystoOr1	ITS-1	PV259768		Oral
*Cystoisospora felis*	CystoOr2	ITS-1	PV259769		Oral
*Cystoisospora felis*	CystoKs1	ITS-1	PV259770		Kostanay
*Cystoisospora felis*	CystoKs2	ITS-1	PV259771		Kostanay

**Figure 3:**
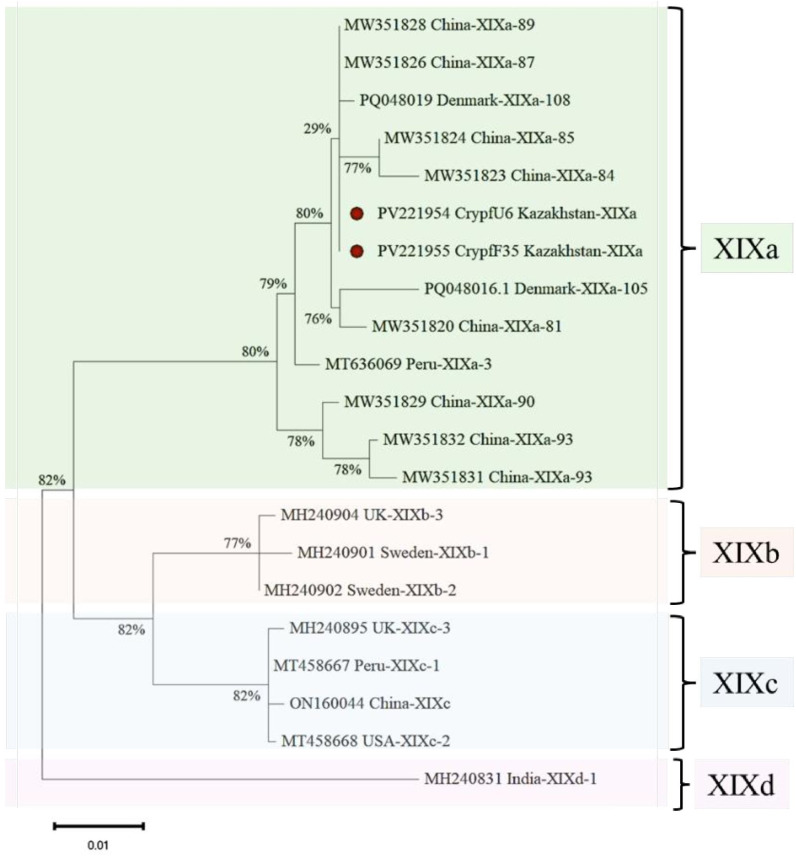
Phylogenetic analysis of XIXa subtypes of *C. felis* based on the gp60 gene sequence. The nucleotide sequences obtained in this study were compared with those of *C. felis* retrieved from GenBank.

*Cystoisospora felis* has been successfully identified in five cities across Kazakhstan. Notably, the cities of Almaty and Kostanay showed no positive samples for either *C. felis* or *C. parvum*, highlighting a significant absence of these parasites in those regions. The **Figure 3** presents a phylogenetic tree illustrating the relationships among *C. felis* XIXa subtypes based on the *gp60* gene sequence. The evolutionary history was inferred by using the Maximum Likelihood method and Tamura-Nei model (Tamura et al., 2021). This analysis involved 21 nucleotide sequences. There were a total of 1332 positions in the final dataset.

**Figure 4:**
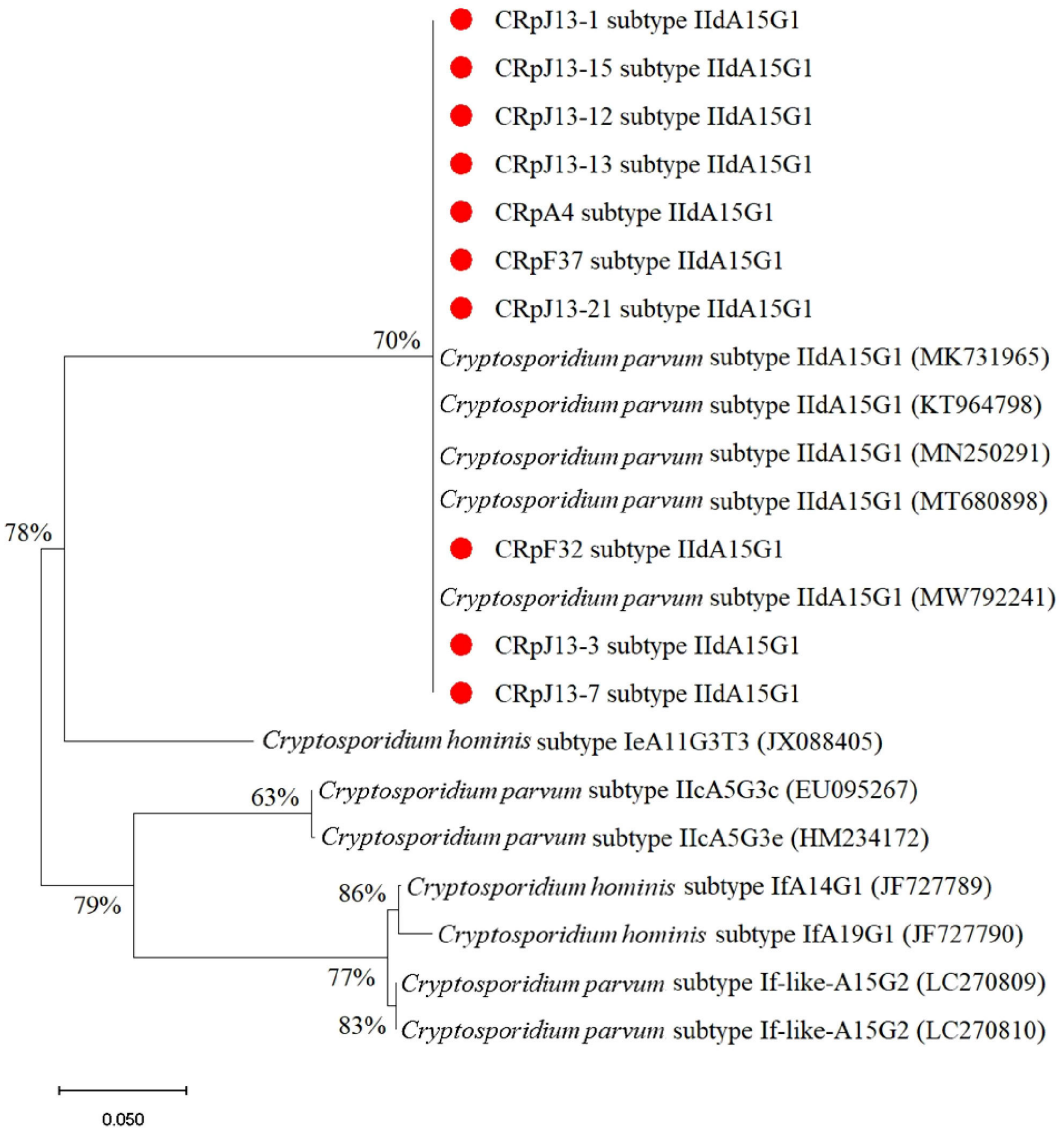
Phylogenetic analysis of subtypes of *C. parvum* based on the gp60 gene sequence. The nucleotide sequences obtained in this study were compared with other subtypes retrieved from GenBank. Red circles indicate new isolates obtained in this study.

**Figure 4** demonstrates that the majority of the studied *C. parvum* isolates obtained in this study belong to subtype IIdA15G1, which is dominant and clusters well with previously reported sequences from different countries on the gp60 gene. This analysis involved 22 nucleotide sequences, the final dataset comprised a total of 1,035 positions.

**Figure 5:**
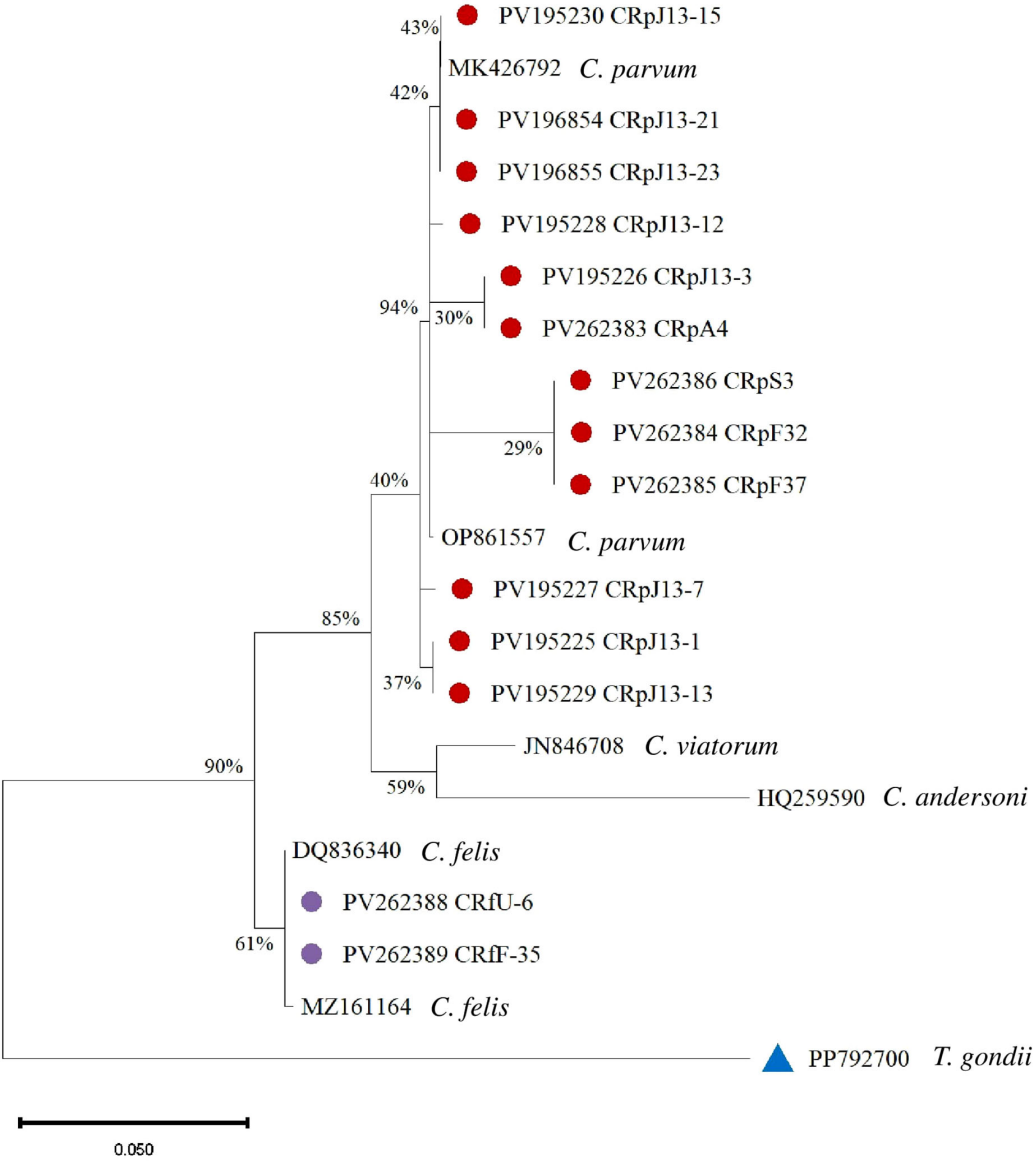
Phylogenetic analysis of *Cryptosporidium* based on the 18S rRNA gene. Isolates from this study are marked with red and purple circles, and a blue triangle represents the outgroup.

*C. parvum* sequences form well-supported clades. The highest support (94%) is observed at one of the internal nodes within the *C. parvum* cluster. *Cryptosporidium viatorum* (JN846708) is a sister group to *C. parvum* with moderate bootstrap support (59%). *Cryptosporidium andersoni* (HQ259590) is more distantly related. *C. felis* sequences (PV262388 CRfU-6 and PV262389 CRfF-35) show 61% bootstrap support (**Figure 5**).

**Figure 6:**
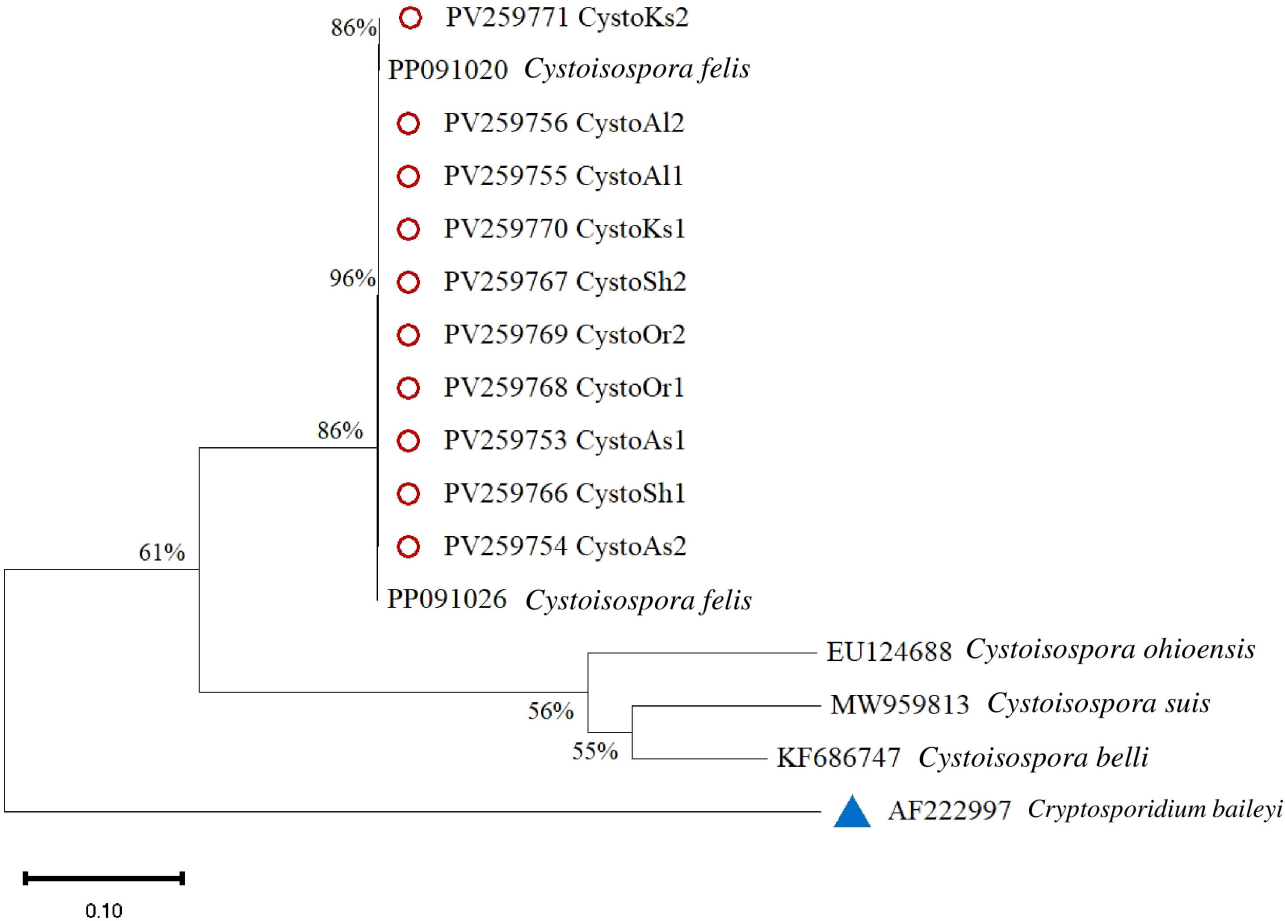
Phylogenetic analysis of *Cystoisospora* based on the ITS-1 gene. The phylogenetic tree was constructed using the maximum likelihood method with 1,000 bootstrap replicates. Divergence = 0.10. Red circles mark isolates from this study, and a blue triangle represents the outgroup.

**Figure 6** illustrates the sequencing and phylogenetic analyses of the amplification products of the ITS-1 region in *Cystoisospora*. A comparative analysis of 10 *Cystoisospora felis* samples was conducted against sequences available in GenBank, revealing a homology range of 86% to 96% compared to the reference isolates. Notably, other species within the *Cystoisospora* genus were distinctly categorized based on these findings. The phylogenetic tree was rooted using *Cryptosporidium baileyi* as an outgroup.

## 4 Discussion

The present study provides significant insights into the molecular epidemiology of *Cryptosporidium* and *Cystoisospora* infections in domestic and shelter cats across five major cities in Kazakhstan (**Figure 1**). The identification of zoonotic species and conserved subtypes suggests both public health relevance and stable transmission patterns within local feline populations.

A total of 1301 fecal samples were examined, with 31 (2.4%) testing positive for *Cryptosporidium* spp. and 121 (9.3%) for *Cystoisospora* sp., and 10 samples were successfully sequenced and confirmed as *Cystoisospora felis* at the species level. *Cryptosporidium parvum* was detected in 12 cases, underscoring its zoonotic potential and public health significance. This pathogen poses a risk not only to immunocompromised individuals but also to immunocompetent populations, particularly children, the elderly, and those exposed to contaminated water or animal contact (Appelbee et al., 2005; Meng et al., 2021; Taghipour et al., 2021).

Meanwhile, the phylogenetic analysis of *Cystoisospora felis* confirmed its close relation to isolates previously reported in different geographical regions (**Figure 5**). The ITS-1 sequencing of *Cystoisospora felis* demonstrated substantial genetic similarity to isolates from each city, indicating a conserved genetic lineage within this species.

The study also revealed key epidemiological patterns. Shelter cats exhibited a higher prevalence of both *Cryptosporidium* (3.1%) and *Cystoisospora* (19.0%) compared to companion cats (1.6% and 5.1%, respectively), likely due to increased exposure to contaminated environments and stress-related immunosuppression. Additionally, younger cats (1-7 months) had a significantly higher infection rate for *Cryptosporidium* (4.5%) and *Cystoisospora* (10.9%) than older age groups, underscoring the susceptibility of juvenile felines. Seasonal variations also played a role, with winter and spring showing the highest infection rates, potentially due to the increased environmental persistence of oocysts in colder temperatures. Environmental studies confirm that oocyst survival is significantly longer in soils and waters kept at lower temperatures (Peng et al., 2008; Li et al., 2010). Epidemiological data in cats show a higher prevalence in winter, reinforcing how seasonal conditions influence transmission (Armon et al., 2016).

A global meta-analysis by Taghipour et al. (2021) reported an overall prevalence of *Cryptosporidium* in cats at 4.0%, with higher rates in shelter and stray populations compared to household pets, aligning with the elevated rates seen in shelter cats in Kazakhstan (Taghipour et al., 2021). In South Korea, Yun et al. (2023) found *Cryptosporidium* spp. in 3.6% of cats, with *Cystoisospora* spp. detected in 7.6%, similar to the Kazakhstan findings for *Cystoisospora felis*. Additionally, the prevalence of *Cryptosporidium* is reported to be 6.5% in shelter cats versus 1.8% in pet cats, with higher rates observed in animals under one year of age (Yun et al., 2023), which confirms our findings in this work. Mendoza and Otranto (2023) also emphasized that younger animals are more vulnerable due to immature immune systems and shelter environments pose a greater risk for protozoan infections due to increased exposure and stress-immunosuppression (Mendoza and Otranto, 2023).

In this study, 12 case of *C. parvum* infection was detected in stray and companion cats. The presence of *C. parvum* in domestic cats poses potential zoonotic risks, especially for individuals with compromised immune systems (Jiang et al., 2020). Molecular characterization of studied *C. felis* isolates (2/14) provided critical insights into the genetic structure of the detected parasites. Notably, the *C. felis* isolates identified in this study belonged to the XIXa subtype, which has been reported globally, reinforcing the genetic homogeneity of this subtype (Lucio-Forster et al., 2010; Xiao and Feng, 2017; Rojas-Lopez et al., 2020; Mendoza and Otranto, 2023). The gp60 subtyping of *C. felis* revealed sequence variations that align with global reports, contributing to the growing database of *Cryptosporidium* genetic diversity (de Oliveira et al., 2021). Of the 12 *Cryptosporidium* isolates analyzed in this study, 10 were successfully sequenced, and all were identified as belonging to the IIdA15G1 subtype. As illustrated in **Figure 4**, these isolates cluster within the *C. parvum* IIdA15G1 clade, which also includes reference sequences retrieved from GenBank (accession numbers: MK731965, KT964798, MN250291, MT680898, MW792241). The bootstrap support for this clade ranges from 70% to 78%, reflecting a robust phylogenetic relationship among isolates of this subtype. In contrast, reference isolates of the *C. hominis* lineage comprising subtypes IeA11G3T3, IfA14G1, and IfA19G1 form a clearly distinct and well-supported branch (bootstrap up to 86%), demonstrating clear genetic separation from *C. parvum*. Additionally, the IIcA5G3c subtype (EU095267, HM234172) and the If-like subtypes (LC270809, LC270810) each form discrete phylogenetic lineages, further underscoring the genetic heterogeneity within *C. parvum*.

These findings underscore the importance of continuous surveillance and molecular monitoring of *Cryptosporidium* and *Cystoisospora* infections in felines, particularly in regions with close human-animal interactions. Given the zoonotic potential of *C. parvum*, targeted public health interventions, including improved hygiene practices and regular veterinary screenings, are essential to mitigate transmission risks. Future studies should explore the role of additional host factors and environmental conditions influencing infection dynamics, as well as assess the broader public health implications of *C. felis* in humans.

Overall, this study contributes to the understanding of unicellular parasite infections in cats in Kazakhstan, emphasizing the need for integrated One Health approaches to monitor and control these infections in both animal and human populations.

## Data Availability

The datasets presented in this study can be found in online repositories. The names of the repository/repositories and accession number(s) can be found in the article/supplementary material.
